# Ophiopogon Polysaccharide Liposome Regulated the Immune Activity of Kupffer Cell through miR-4796

**DOI:** 10.3390/ijms232314659

**Published:** 2022-11-24

**Authors:** Jing Cui, Xingxue Pan, Xueqin Duan, Liting Ke, Xiaoping Song, Weimin Zhang, Wuren Ma, Yingqiu Liu, Yunpeng Fan

**Affiliations:** College of Veterinary Medicine, Northwest A&F University, Yangling, Xianyang 712100, China

**Keywords:** ophiopogon polysaccharide liposome, miR-4796, Kupffer cells, immune activity

## Abstract

The purpose of this article is to study the effects and mechanism of miR-4796 in the process of ophiopogon polysaccharide liposome (OPL) regulation of the immune activity of Kupffer cells (KCs). In this study, KCs were used as cell models, and were treated with OPL in different concentrations after being transfected with miR-4796 mimic or miR-4796 inhibitor. Firstly, the secretion of NO and iNOS, phagocytic activity, the expression of surface molecules CD14 and MHC II, apoptosis and ROS secretion were measured by Griess, flow cytometry, fluorescence staining and ELISA. Then, real-time PCR and Western blot were used to measure the expression of TLR4, IKKβ, MyD88 and NF-κB in the TLR4-NF-κB signaling pathway. The results showed that after transfection with miR-4796 mimic, the secretion of NO and iNOS, cell migration, cell phagocytosis and expression levels of CD14 and MHC II in the OPL group were significantly higher than those in the miR-4796 mimic control group (*p* < 0.05; *p* < 0.01). In addition, the mRNA and protein expression levels of TLR4, MyD88 and NF-κB were significantly higher than those in miR-4796 mimic control group (*p* < 0.05; *p* < 0.01). After transfection with miR-4796 inhibitor, the secretion of NO and iNOS, cell migration, cell phagocytosis, expression of CD14 and MHCII in OPL group were significantly higher than those in the miR-4796 inhibitor control group (*p* < 0.05; *p* < 0.01). These results indicated that OPL could regulate the immune activity of KCs by regulating miR-4796 and activating the TLR4-NF-κB signaling pathway.

## 1. Introduction

Ophiopogon is the dried radix of *Ophiopogon japonicus* (F.L) Ker-Gawl of Liliaceae, which was first described in Shen Nong’s Herbal Classic. It has the effect of nourishing Yin and promoting the production of body fluids, moistening the lungs and clearing the heart, and is used to treat pulmonary dryness with dry cough, thirst caused by deficiency of body fluid, and constipation due to intestinal dryness [1]. It has been reported that, as one of the main active components of Ophiopogon, Ophiopogon polysaccharide (OP) has a variety of biological activities, mainly focused on enhancing immunity, anti-allergy and anti-viral effects, and so on. OP is the effective part of ophiopogon exerting the immune-enhancing effect [2]. Studies have revealed that effective immune stimulants come from polysaccharides of natural medicines, and the increase in NO level and the endocytosis of the polysaccharide may provide new ideas for improving innate response and immunotherapy [3].

Some studies have shown that OP could conspicuously increase the weight of the thymus and spleen in young mice, indicating that OP may promote the proliferation of T lymphocytes and B lymphocytes, which has a positive effect on cellular and humoral immunity. At the same time, it can also enhance the phagocytosis ability of the reticuloendothelial system of mice and increase the content of hemolysin in serum, which indicates that OP can significantly regulate the humoral immune function of the body. As a result, OP has good immune enhancing and stimulating effects [4]. Li Ming [5] explored the effects of OP on the immune function and antioxidant capacity of rats under long-term load training, and found that OP could improve its immune function, inhibit peroxidation damage and glycogen depletion, which had the better development prospect. The results showed that the supplementation of OP in the diet could increase the average daily gain, average daily feed intake, reduce the ratio of feed to gain, and raise the antibody titer of Newcastle disease and serum IgA, IgG and IgM concentrations of black bone chicken male chicks. Interestingly, 1% OP could promote the growth performance of the black bone chickens and adjust the immunosuppression caused by cyclophosphamide to normal levels [6].

Liposome, which is an effective drug carrier, is composed of one or more phospholipid bilayers of spherical vesicles. In addition, the bubble of the biological membrane structure has lots of advantages, such as low toxicity, good biocompatibility, and a lack of any immunosuppressive effect, which can improve the stability of the contained substances, protect drugs targeting certain organs or tissue, and enhance the therapeutic efficacy of drugs. In recent years, the study of drugs encapsulated by liposome has become a research hotspot. Liposomes have been continuously improved and optimized since their development. At present, the development of liposomes as drug carriers and as adjuvant preparations for vaccines is still a research hotspots [7,8,9,10]. Based on some studies, licorice polysaccharide liposome could significantly promote the proliferation of immature chBM-DCs, enhance the ability of mature chBM-DCs to induce T cell proliferation, and regulate the secretion of cytokines such as IL-2, IFN-γ and IL-10 by mature chBM-DCs. It was proved that licorice polysaccharide encapsulated by liposome could significantly improve its immunoregulatory activity [11].

MicroRNA (miRNA) is a kind of small endogenous non-coding RNA with a length of about 22 to 26 nucleotides. By binding to the direct complementary sequence of the 3′ untranslated region (3′ UTR) of mRNAs, microRNA controls post-transcriptional gene silencing, thereby targeting them for translational inhibition or degradation. As a small non-coding RNA molecule with endogenous gene expression regulation, miRNA binding to target gene mRNA can regulate the expression of the target gene at the post-transcriptional level and further participate in the development, metabolism and other pathological and physiological processes of organisms. For example, miRNA-155 is highly expressed in activated lymphocytes and is involved in the immune regulation process of various lymphocyte subsets, including B cells, CD8^+^T and CD4^+^T cells, helper T cell type 1 (Th1), Th2, Th17 and regulatory T cells (Treg), which can affect the differentiation direction of T cells and regulate their immune response activity. Thus, the body’s autoimmunity, tumor immunity, anti-infection immunity and other important immune processes are affected [12,13,14]. In recent years, the research and development of low-toxicity and high-efficiency immune enhancers has been of great significance to the research and development of vaccines, especially new vaccines, which are affected by many factors, such as widespread use of vaccines, high cost and poor response to vaccination [15]. The results of some recent studies revealed that polysaccharides and liposomes have been developed and widely used in clinical practice as vaccine adjuvants [16]. Moreover, a great deal of studies have shown that Traditional Chinese Medicine (TCM) polysaccharide encapsulation by liposomes could enhance its immune activity, but its mechanism of action is still unclear. At the same time, a large number of studies have proven that miRNA plays an important role in various biological processes [17]. Therefore, the relationship between the regulation of immune activity by TCM polysaccharide liposome and miRNA has become a research hotspot. In order to lay the foundations for the development of new types of TCM immune enhancers, this paper further explore the mechanism of immune effect enhancement by TCM polysaccharide liposomes.

In the previous experiment, it was proven that Ophiopogon polysaccharide liposome (OPL) possessed the better immune enhancement activities in vitro and in vivo, and the effect was significantly higher than that of OP [18,19,20]. Subsequent research has found that microRNA-4796 (miR-4796) was associated with OPL immunoregulatory activity, which was screened out by high-throughput sequencing. The mature sequence of miR-4796 is AAGTTGCAGGGTATAGAC. However, how does OPL regulate miR-4796 expression during immune regulation? What role does miR-4796 play in the process of OPL immune regulation? The answers to these questions are unclear, and still need further study. In this experiment, Kupffer cells (KCs) were selected as the model, and the effect of miR-4796 on OPL regulating the immune activity of KCs was studied by fluorescence staining, flow cytometry, Griess and other methods; the aim was to further explore the mechanism of OPL on immune activity regulation, and to provide certain theoretical basis for the development and utilization of new Chinese medicine immune enhancers.

## 2. Results

### 2.1. The Expression of miR-4796 after Transfected miR-4796 Mimic or Inhibitor 

**Figure 1:**
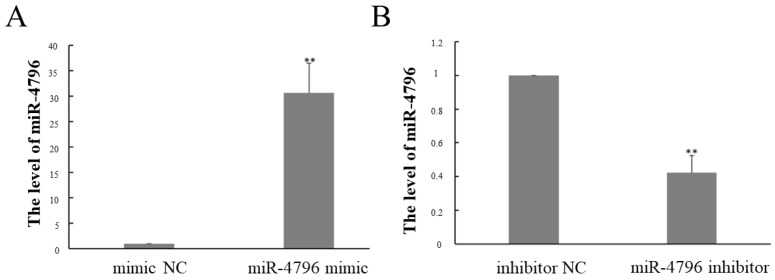
Expression level of miR-4796. (**A**): Expression level of miR-4796 transfected with miR-4796 mimic; (**B**): Expression level of miR-4796 transfected with miR-4796 inhibitor. Each value represents the mean ± SD (*n* = 3). ** *p* < 0.01 compared with the NC group.

The expression of miR-4796 was shown in Figure 1. After transfected miR-4796 mimic, the expression of miR-4796 was significantly increased compared with the inhibitor NC group (*p* < 0.05) (Figure 1A). The expression of miR-4796 was significantly reduced after transfected miR-4796 inhibitor group (*p* < 0.05) (Figure 1B).

### 2.2. The Effect of OPL on the Secretion of NO and iNOS

**Figure 2:**
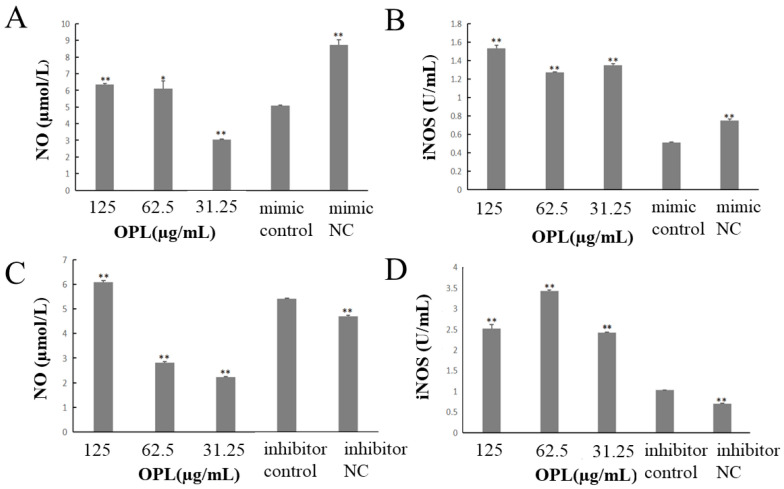
Influence of OPL on NO and iNOS contents. (**A**): Expression level of NO transfected with miR-4796 mimic; (**B**): expression level of iNOS transfected with miR-4796 mimic; (**C**): expression level of NO transfected with miR-4796 inhibitor; (**D**): expression level of iNOS transfected with miR-4796 inhibitor. Each value represents the mean ± SD (*n* = 3). * *p* < 0.05, ** *p* < 0.01 compared with the control group.

The results were shown in Figure 2. The contents of NO and iNOS in mimic control group were significantly lower than mimic NC group (*p* < 0.01) (Figure 2A,B) after transfecting miR-4796 mimic. Compared with miR-4796 mimic control group, the content of NO in OPL group at 125 and 62.5 μg/mL was significantly reduced (*p* < 0.01, *p* < 0.05) (Figure 2A), and the content of iNOS in OPL group at 125–31.25 μg/mL was significantly increased (*p* < 0.01) (Figure 2B). After transfecting miR-4796 inhibitor, the content of NO and iNOS in inhibitor control group was significantly higher than that in the inhibitor NC group (*p* < 0.01) (Figure 2C,D). Compared with the miR-4796 inhibitor control group, the content of NO in the OPL group at 125 μg/mL was significantly reduced (*p* < 0.01) (Figure 2C), and the content of iNOS in the OPL group at 125–31.25 μg/mL was significantly increased (*p* < 0.01) (Figure 2D). The results showed that the secretion of NO and iNOS was significantly decreased after transfecting mir-4796 mimic. After transfected mir-4796 inhibitor, the secretion of iNOS was significantly increased. OPL could promote the secretion of NO and iNOS after transfection of mir-4796 mimic or mir-4796 inhibitor.

### 2.3. The Effect of OPL on Cell Migration

**Figure 3:**
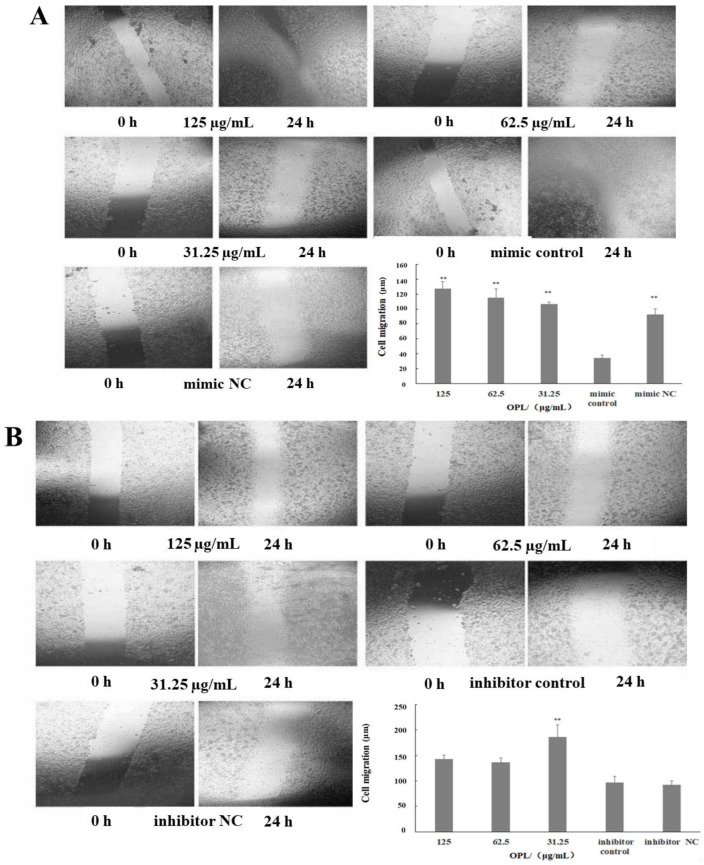
The effect of OPL on cell migration. (**A**): Cell migration in the transfected miR-4796 mimic group (×40); (**B**): cell migration in the miR-4796 inhibitor transfected group (×40). Each value represents the mean ± SD (*n* = 3). ** *p* < 0.01 compared with control group.

The results are illustrated in Figure 3. After transfected miR-4796 mimic, the cell migration in the miR-4796 mimic control group was significantly lower than the mimic NC group (*p* < 0.01). Compared with the mir-4796 mimic control group, the cell migration in the OPL group at 125–31.25 μg/mL was significantly increased (*p* < 0.01) (Figure 3A). After transfecting miR-4796 inhibitor, the cell migration in the inhibitor control was no different from the inhibitor NC group. The cell migration in the OPL group at 31.25 μg/mL was significantly higher than that in miR-4796 inhibitor control group (*p* < 0.01) (Figure 3B). The results showed that cell migration ability was inhibited after transfecting miR-4796 mimic. Furthermore, OPL could promote the cell migration ability after transfecting miR-4796 mimic.

### 2.4. The Effect of OPL on the Phagocytosis of KCs for FITC-Dextran by Fluorescence Staining

**Figure 4:**
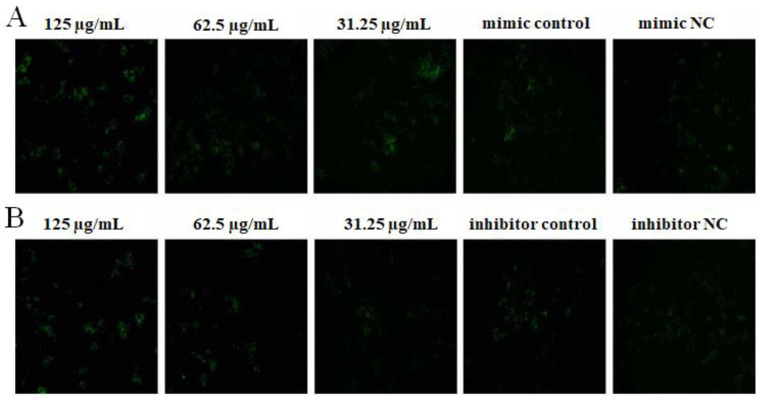
The effect of OPL on the phagocytosis of FITC-Dextran. (**A**): The group transfected with miR-4796 mimic phagocytosis of FITC-Dextran (×200); (**B**): the group transfected with miR-4796 inhibitor phagocytosis of FITC-Dextran (×200).

The results are illustrated in Figure 4. After transfecting miR-4796 mimic, there was no significant difference for the green fluorescence between the mimic control group and the mimic NC group. The green fluorescence in the OPL group at 125 and 31.25 μg/mL was more than the mimic control group (Figure 4A). After transfecting miR-4796 inhibitor, there was no significant difference for the green fluorescence between the inhibitor control group and the inhibitor NC group. Green fluorescence in the OPL group at 125 and 62.5 μg/mL was more than that in the miR-4796 inhibitor control (Figure 4B). The results indicated that there was no change in phagocytosis after transfecting miR-4796 mimic and inhibitor. OPL could promote the phagocytic activity of KCs on FITC-dextran after transfecting miR-4796 mimic and inhibitor.

### 2.5. The Effect of OPL on the Phagocytosis of KCs for FITC-Dextran by Flow Cytometry

**Figure 5:**
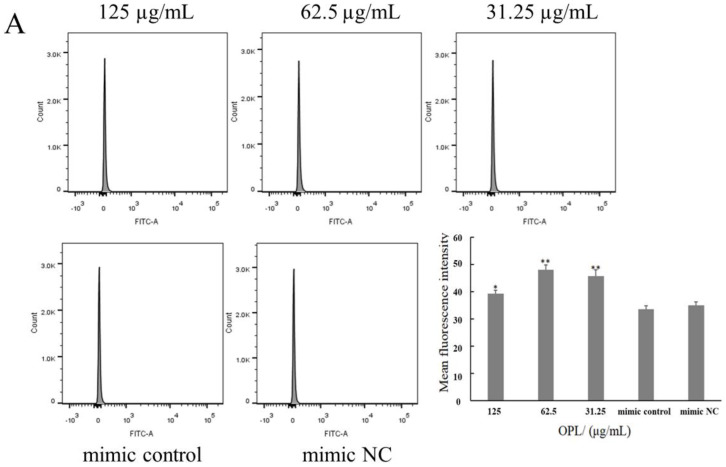

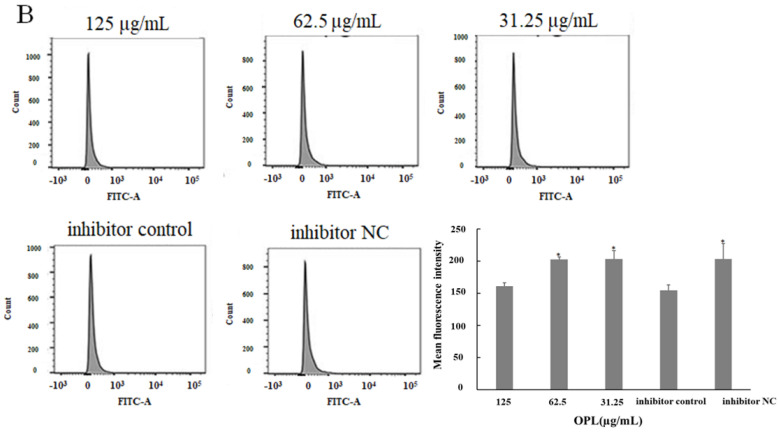
The effect of OPL on the phagocytosis of FITC-Dextran. (**A**): Average fluorescence intensity of transfected miR-4796 mimic group; (**B**): average fluorescence intensity in the transfected miR-4796. Each value represents the mean ± SD (*n* = 3). * *p* < 0.05, ** *p* < 0.01 compared with the control group.

The results of the flow cytometry analysis are shown in Figure 5. After transfecting miR-4796 mimic, there was no significant difference in the average fluorescence intensity between the mimic control group and the mimic NC group. The average fluorescence intensity in the OPL group at 125–31.25 μg/mL groups was significantly higher than that in the mimic control (*p* < 0.05) (*p* < 0.01) (Figure 5A). After transfecting miR-4796 inhibitor, the average fluorescence intensity in the mimic control group was significantly lower than in the mimic NC group (*p* < 0.05). The average fluorescence intensity in the OPL group at 62.5 and 31.25 μg/mL was significantly higher than that in the inhibitor control group (*p* < 0.05) (Figure 5B). The results showed that OPL could increase the phagocytic activity of KCs after transfecting miR-4796 mimic and inhibitor.

### 2.6. The Effect of OPL on the Phagocytosis of KCs for FITC-OVA

**Figure 6:**
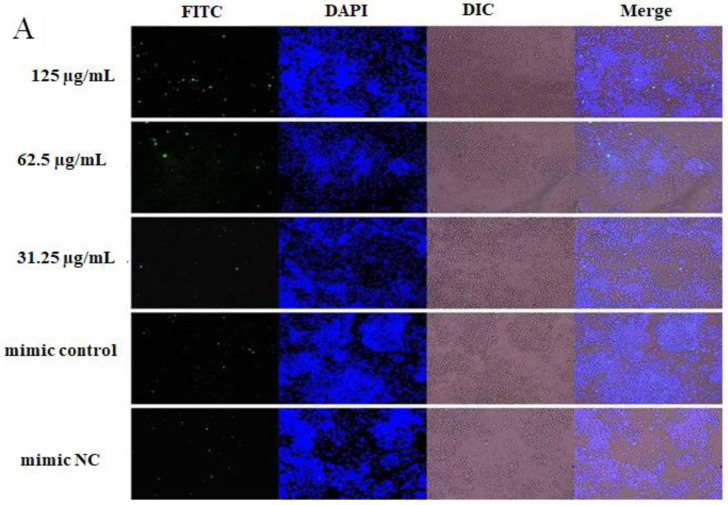

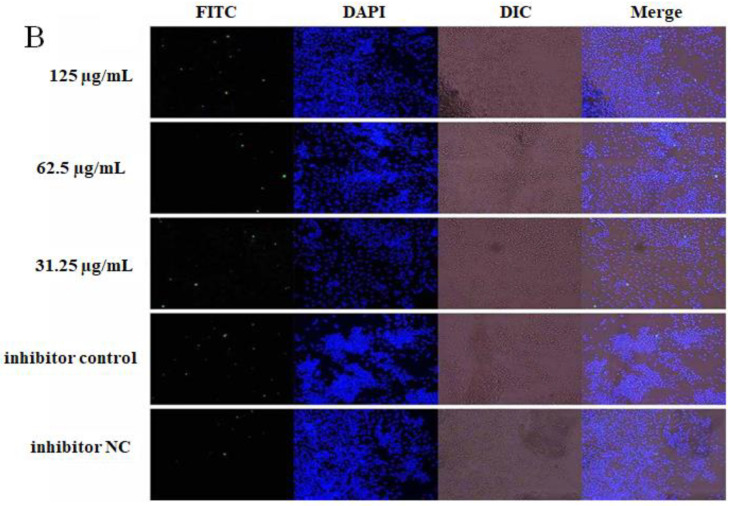
The effect of OPL on phagocytosis of FITC-OVA. (**A**): The group transfected with miR-4796 mimic phagocytosis of FITC-OVA (×200); (**B**): the group transfected with miR-4796 inhibitor phagocytosis of FITC-OVA (×200).

The results are illustrated in Figure 6. After transfecting miR-4796 mimic, the green fluorescence of the miR-4796 mimic control group was not significantly different from that in the mimic NC group, and the green fluorescence in the OPL group at 125 and 62.5 μg/mL was more than that in the miR-4796 mimic control (Figure 6A). After transfecting miR-4796 inhibitor, there was no significant difference in green fluorescence among the inhibitor control group, inhibitor NC group and OPL group (Figure 6B). The results showed that OPL could increase the phagocytic activity of KCs on FITC-OVA after transfecting miR-4796 mimic.

### 2.7. The Effect of OPL on the Cell Apoptosis

**Figure 7:**
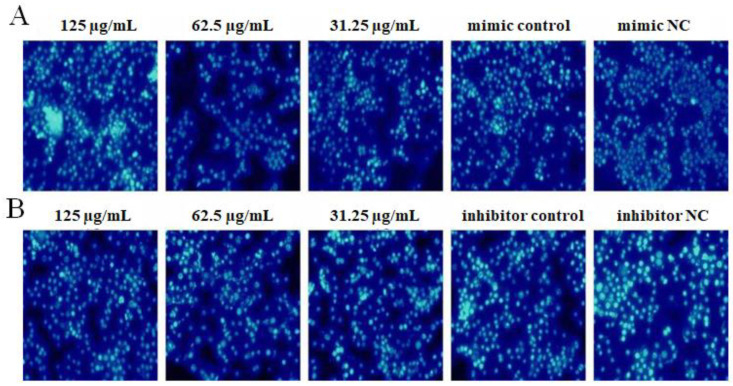
The effect of OPL on cell apoptosis. (**A**): Apoptosis level in the transfected miR-4796 mimic group (×200); (**B**): apoptosis level in the transfected miR-4796 inhibitor group (×200).

The results are shown in Figure 7. After transfecting miR-4796 mimic, the cell nucleus in the miR-4796 mimic control group was denser than that in the mimic NC group. Compared with the mimic control, cells in the OPL group at 62.5 and 31.25 μg/mL had the best conditions and only a small part of the nucleus was whiter (Figure 7A). After transfecting with miR-4796 inhibitor, the cell nucleus in the miR-4796 inhibitor control group was denser than that in the inhibitor NC group. Compared with the miR-4796 inhibitor control, cells in the OPL groups at 125 and 62.5 μg/mL possessed the best morphology and demonstrated less nuclear bleaching (Figure 7B). The results indicated that the cell apoptosis rate increased after transfecting miR-4796 mimic and miR-4796 inhibitor. In addition, OPL had a significant inhibitory effect on cell apoptosis after transfecting with miR-4796 mimic and inhibitor.

### 2.8. The Effect of OPL on the Expression of CD14 and MHCII

**Figure 8:**
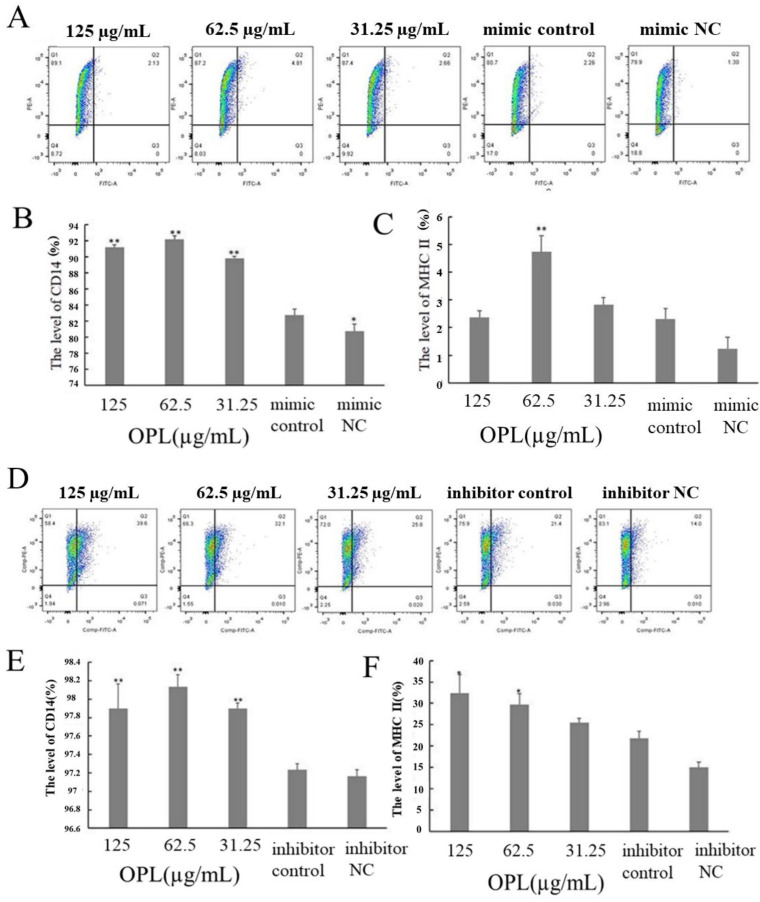
The expression of CD14 and MHC II. (**A**): Flow cytometric analysis of cells transfected with miR-4796 mimic; (**B**): expression level of CD14 in transfected miR-4796 mimic group; (**C**): expression level of MHC II in transfected miR-4796 mimic group; (**D**): flow cytometric analysis of cells transfected with miR-4796 inhibitor; (**E**): expression level of CD14 in transfected miR-4796 inhibitor group; (**F**): expression level of MHC II in transfected miR-4796 inhibitor group. Each value represents the mean ± SD (*n* = 3). * *p* < 0.05, ** *p* < 0.01 compared with the control group.

The results are illustrated in Figure 8. After transfecting miR-4796 mimic, the expression of CD14 (Figure 8B) in the mimic control group was significantly higher than that in the mimic NC group. The expression of CD14 (Figure 8B) in the OPL group at 125–31.25 μg/mL (*p* < 0.01) and MHCII (Figure 8C) in the OPL group at 62.5 μg/mL (*p* < 0.01) was significantly higher than that in the miR-4796 mimic control. After transfecting miR-4796 inhibitor, there was no significant difference in the expression of CD14 and MHCII between the inhibitor control group and the inhibitor NC group. The expression of CD14 (*p* < 0.01) (Figure 8E) in the OPL group at 125–31.25 μg/mL and MHCII (*p* < 0.05) (Figure 8F) in the OPL group at 125–62.5 μg/mL was much higher than that in miR-4796 inhibitor control. The results showed that the expression of CD14 could be promoted after transfecting miR-4796 mimic. After transfecting, neither miR-4796 mimic nor inhibitor could affect the expression of MHCII. OPL could promote the expression of CD14 and MHCII after transfecting miR-4796 mimic and inhibitor.

### 2.9. The Effect of OPL on the Secretion of ROS

**Figure 9:**
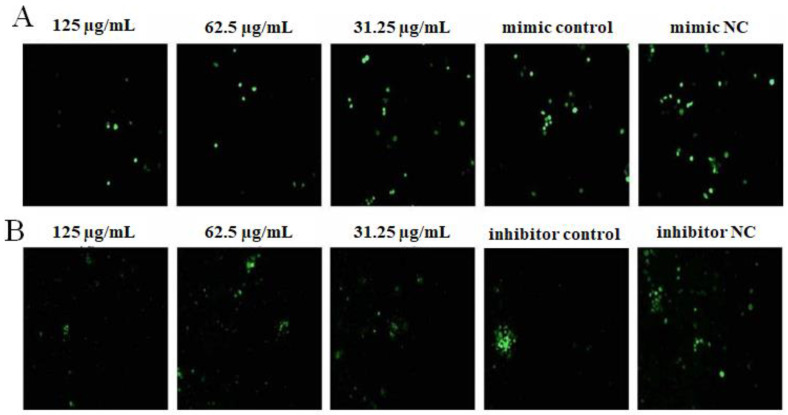
The effect of OPL on ROS secretion. (**A**): ROS level in the transfected miR-4796 mimic group (×200); (**B**): ROS level in the miR-4796 inhibitor transfection group (×200).

The results are shown in Figure 9. After transfecting miR-4796 mimic, there was no significant difference between the miR-4796 mimic control group and the mimic NC group. Green fluorescence in the OPL group at 125–62.5 μg/mL was lower than that in the miR-4796 mimic control (Figure 9A). After transfecting miR-4796 inhibitor, green fluorescence in the miR-4796 inhibitor control was lower than that in the miR-4796 inhibitor NC group. Furthermore, green fluorescence in the OPL group at 125–31.25 μg/mL was lower than that in the miR-4796 inhibitor control (Figure 9B). The results indicated that the secretion of ROS could not be affected after transfecting miR-4796 mimic, but secretion could be inhibited after transfecting miR-4796 inhibitor. OPL could inhibit the secretion of ROS after transfecting miR-4796 mimic and inhibitor.

### 2.10. The Effect of OPL on the mRNA and Protein Expression of TLR4, MyD88, IKKβ and NF-κB

**Figure 10:**
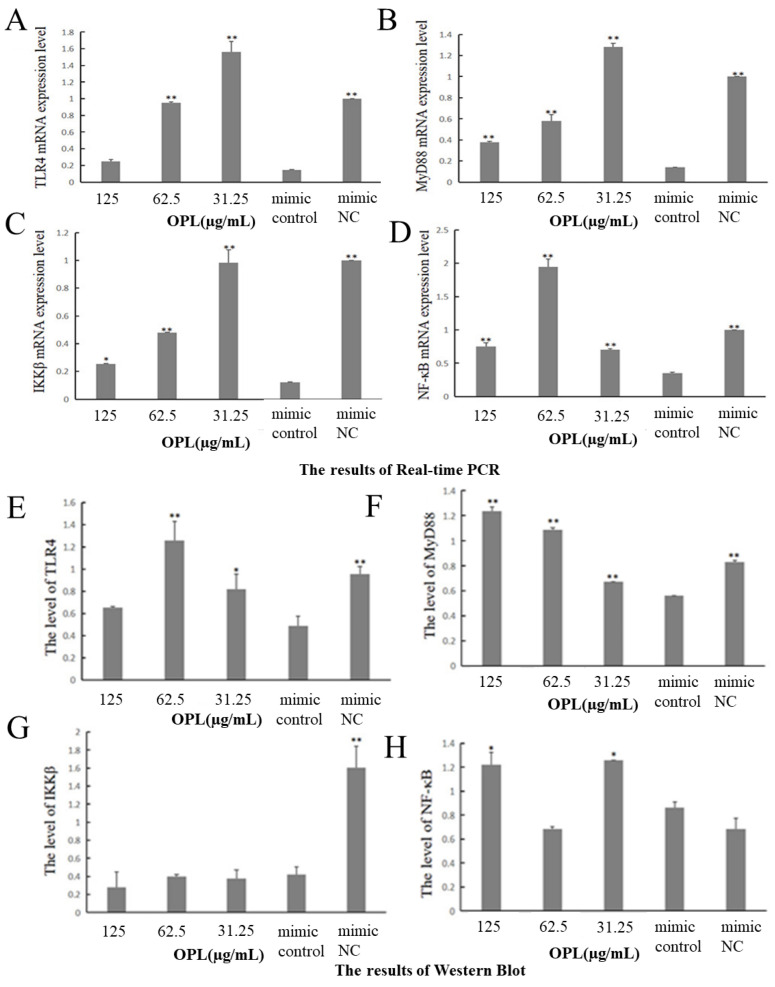

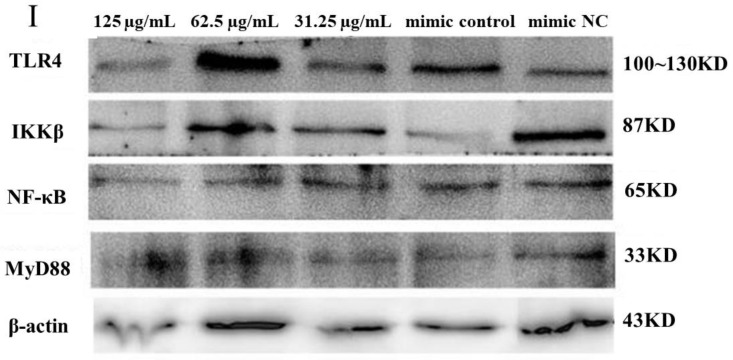
Influence of OPL on mRNA and protein expression after transfection with miR-4796 mimic. (**A**): TLR4 mRNA expression level; (**B**): MyD88 mRNA expression level; (**C**): IKKβ mRNA expression; (**D**): NF-κB mRNA expression; (**E**): TLR4 protein expression level; (**F**): MyD88 protein expression level; (**G**): IKKβ protein expression level; (**H**): NF-κB protein expression level; (**I**): protein bands. Each value represents the mean ± SD (*n* = 3). * *p* < 0.05, ** *p* < 0.01 compared with the control group.

The results are illustrated in Figure 10. After transfecting miR-4796 mimic, the mRNA expressions of TLR4 (Figure 10A), MyD88 (Figure 10B), IKKβ (Figure 10C) and NF-κB (Figure 10D) in the miR-4796 mimic control group were significantly lower than that in the mimic NC (*p* < 0.01). Compared with miR-4796 mimic control, OPL at 62.5 and 31.25 μg/mL significantly promoted the mRNA expression of TLR4 (*p* < 0.01) (Figure 10A), while OPL at 125–31.25 μg/mL promoted the mRNA expression of MyD88, IKKβ and NF-κB (*p* < 0.05) (*p* < 0.01) (Figure 10B–D). 

After miR-4796 mimic was transfected, the protein expressions of TLR4 (Figure 10E), MyD88 (Figure 10F) and IKKβ (Figure 10G) in the miR-4796 mimic control group were significantly lower than that in the mimic NC (*p* < 0.01). Compared with the miR-4796 mimic control, OPL significantly promoted the expression of TLR4 at 62.5 and 31.25 μg/mL (*p* < 0.05) (*p* < 0.01) (Figure 10E), promoted the expression of MyD88 at 125–31.25 μg/mL (*p* < 0.01) (Figure 10F), and promoted the expression of NF-κB at 125 and 31.25 μg/mL (Figure 10H) (*p* < 0.05).

## 3. Discussion

As intrinsic hepatic macrophages, KCs are derived from bone marrow monocytes, which account for about 80~90% of the total number of macrophages in the human body and about 15% of the total number of hepatocytes. Based on some studies, common drug liposomes are mainly ingested by mononuclear macrophages (especially KCs) in the liver and spleen reticuloendothelial system after entering the body, thereby activating the body’s autoimmune function [21]. Therefore, KCs were selected as the cell model in this study.

NO, which is an important second messenger molecule in mammals, has a wide range of biological functions. iNOS, a non-calcium-dependent synthetase, synthesizes NO mainly through the L-arginine pathway [22]. Studies have shown that traditional Chinese medicine and its active components could regulate the secretion of NO and iNOS by regulating miRNAs. For example, Panax notoginseng saponin could regulate the expression of IRF-1, which had a regulatory effect on the secretion of NO and iNOS by regulating the expression of miR-23a [23]. The results of this study showed that transfection of miR-4796 mimic could inhibit the secretion of NO or iNOS, and transfection of miR-4796 inhibitor could promote the secretion of NO or iNOS; these findings are similar to the study results relating to miR-21. These results indicated that miR-4796 could regulate the secretion of NO and iNOS, and OPL could promote the secretion of NO and iNOS by down-regulating the expression of miR-4796.

A cell scratch test can observe the migration ability of cells by calculating the migration distance of cells [24]. Some studies have concluded that miRNAs could regulate the migration level of cells, and traditional Chinese medicine or its active components could affect the migration ability of cells by regulating miRNAs. For example, the expression of miR-151 in HepG2 cells could be inhibited by the polysaccharide from roots of Radix Tetrastigma (RTP). The overexpression of miR-151 could reverse the effects of RTP on the proliferation, apoptosis, migration and invasion of HepG2 cells, which indicated that RTP might affect the biological behavior of HepG2 cells by down-regulating the expression of miR-151 [25]. In this study, the cell scratch test was used to observe the migration ability of the cells by calculating the migration distance of the cells. The results showed that OPL could enhance the migration ability of the cells transfected with miR-4796 mimic and miR-4796 inhibitor, which was similar to the test results of the RTP.

Phagocytosis is one of the most important functions of KCs. Phagocytosis refers to the phagocytosis of a variety of pathogenic factors, such as pathogens, by means of the folds on the surface of the cell membrane and the extended pseudopodia, and the elimination of pathogens by a variety of hydrolytic enzymes in the lysosome. It is one of the most important ways for the body to exert non-specific immunity [26]. Studies have shown that miRNAs could affect phagocytosis among cells. However, whether OPL could improve the phagocytic activity of cells by regulating miRNAs is still unclear. Therefore, in order to verify whether OPL could affect the phagocytic function of cells by regulating miR-4796, three different methods—FITC-dextran cells by fluorescence staining, FITC-dextran cells by flow cytometry, and FITC-OVA by fluorescence staining—were used to observe the phagocytosis in this study. The results revealed that OPL could promote the phagocytosis of KCs after transfection with miR-4796 mimic. Studies have proven that miR-34c could promote the phagocytosis of crystals by macrophages [27], which is consistent with the results of this study.

Fluorescent dye Hoechst 33,258 is often used to detect apoptosis. Hoechst 33,258 enter normal cells with difficulty, while due to enhanced membrane permeability, chromosomal DNA structure changes and other reasons, apoptotic cells facilitate the accumulation of Hoechst 33,258 in cells, so that the fluorescence effect is stronger than in normal cells. Research has shown that traditional Chinese medicine and its active components could regulate the apoptosis of cells by regulating miRNAs. For example, *Lycium barbarum* Polysaccharide (LBP) could inhibit the apoptosis of H9c2 cells by regulating the expression of miR-122 [28]. Furthermore, astragalus polysaccharide can reduce the apoptosis of neural stem cells in rats by regulating miR-138 to reduce oxidative damage to cells [29]. The results of this study showed that transfection of miR-4796 mimic promoted cell apoptosis, and transfection of miR-4796 inhibitor inhibited cell apoptosis, which was similar to the research results of miR-21. In addition, the experimental results also proved that OPL could inhibit cell apoptosis after transfection with miR-4796, indicating that OPL could inhibit cell apoptosis by down-regulating the expression of miR-4796, which was similar to the study results of LBP and astragalus polysaccharide.

CD14 is a leukocyte differentiation antigen with a molecular weight of about 55 kD on the surface of monocytes and other cells [30]. It is a glycoprotein that plays an important role in the occurrence, development and prognosis of diseases. MHC II is located on the surface of antigen-presenting cells, such as macrophages, monocytes and so on. KCs is the main cells that induce lymphocyte proliferation, and this induction is limited by MHC II molecules [31]. Research has shown that miRNAs are involved in the regulation of CD14 expression; for instance, transfection of miR-146a-5p mimic up-regulates the expression level of CD14, and transfection of miR-146a-5p inhibitor down-regulates the expression level of CD14 [32]. The results of this study showed that OPL could promote the expression of CD14 or MHC II after miR-4796 transfection, indicating that OPL could regulate the expression of CD14 or MHC II through miR-4796.

ROS is considered to be a toxic product of cell metabolism, mainly produced by mitochondria in most animal cells and involved in the oxidative response during cell stress as signaling molecules [33]. ROS is also a key mediator of the MAPK and NF-κB pathways in immune responses [34]. Studies have proven that miRNAs could regulate ROS secretion; for example, overexpression of miR-155 could promote ROS secretion [35], and studies have proved that traditional Chinese medicine and its active components could affect ROS secretion by regulating the expression of miRNAs [36,37]. The results of this study showed that transfection with miR-4796 inhibitor inhibited ROS secretion, which was similar to that of miR-155. In addition, OPL was proved to inhibit the secretion of ROS after transfection with miR-4796 mimic and miR-4796 inhibitor, indicating that OPL could regulate ROS secretion through miR-4796.

NF-κB transcription factor is a protein complex that regulates DNA transcription. It consists of P50 (NF-κB1), RelA (P65) or p52 (NF-κB2) and RelB. It is the NF-κB signaling pathway that plays a key role in maintaining normal host physiology. In order to avoid unwanted immune responses, NF-κB signaling is usually inactive [38]. Traditional Chinese medicine and its active components can activate this pathway through various mechanisms to improve the immune activity of the body. For example, Isaria cicadae Miquel polysaccharide can increase expression of the TLR4 protein and promote the phosphorylation of NF-κB. Furthermore, the TLR4-NF-κB signaling pathway is activated to improve immune activity [39]. In this study, four key factors in the NF-κB pathway, TLR4, IKKβ, MyD88 and NF-κB, as well as the downstream inflammatory factor IL-6, were selected for detection. TLR4 can specifically recognize specific molecular structures in pathogens, activate downstream signaling pathways, and finally activate inflammatory cells to produce inflammatory cytokines through the MyD88 classical pathway or the MyD88 non-classical pathway; thus, the first barrier in the body’s immune system is constituted. MyD88 conveys upstream information in the NF-κB signaling pathway and plays an important role in the occurrence and development of diseases. As an IKB kinase, IKKB activates NF-κB into the nucleus by activating the inhibitory protein IKB of NF-κB under the stimulation of external factors, and then regulates the expression of the corresponding genes [40]. NF-κB is an important nuclear transcription factor in cells. It participates in the inflammatory and immune response of the body, and can regulate cell apoptosis and stress response. Research has shown that Traditional Chinese Medicine and its active components could affect the expression levels of TLR4, MyD88 and NF-κB by regulating miRNAs [41,42]. The results of this study also proved that OPL could affect the expression of TLR4, MyD88 and NF-κB mRNA and protein by regulating miR-4796. The results of the Western blot test showed that the expression levels of IKKβ, TLR4 and NF-κB were not consistent. The reason might be that the increased expression levels of TLR4 and MyD88 did not reach the threshold for increasing the expression of IKKβ. The expression levels of TLR4, MyD88 and NF-κB were increased, while the expression levels of IKKβ remained unchanged. There are two reasons for the inconsistent results between mRNA level expression and protein level expression. On the one hand, gene expression could be divided into transcription and translation levels, namely mRNA level and protein level. However, in the process of transcription and translation, eukaryotic gene expression appears spatial and temporal gaps. Secondly, after transcription, post-transcriptional processing, degradation of transcription products, translation, post-translational processing and modification of several levels will occur. On the other hand, protein expression is not only restricted by one gene; a certain protein may be regulated by multiple genes, and on the contrary, a gene might regulate multiple proteins, so the transcription level and translation level are not completely consistent [43,44]. Although the expression of mRNA and proteins are not consistent, the results still revealed that the effect of OPL on mRNA expression and protein was basically the same. After KC transfection with miR-4796 mimic, adding different concentrations of OPL could significantly promote the expression of TLR4, MyD88, NF-κB mRNA and protein.

## 4. Materials and Methods 

### 4.1. Materials

#### 4.1.1. Cell

KCs derived from mice (BNCC340733) were purchased from BeiNa Biological Company, Beijing, China.

#### 4.1.2. Reagent

For the reagent, the following were used: MiR-4796 mimic, miR-4796 inhibitor, mimic negative control (mimic NC) and inhibitor negative control (inhibitor NC) (Guangzhou Ruibo Biotechnology Co., Ltd., Guangzhou, China); Lipofectamine 2000 transfection reagent; antibody FITC-MHC II and CD14 (Thermo Fisher Scientific); Nitrite detection kit and nitric oxide synthase (iNOS) typing kit (Nanjing Jiancheng Biological Engineering Institute, Najing, China); FITC-dextran, Hoechst 33,258 staining solution and FITC-OVA (Beijing Suolaibao Technology Co., Ltd., Beijing, China); ROS detection kit (Biyuntian Biotechnology Co., Ltd., Shanghai, China); 2× Fast qPCR Master Mixture (Green) fluorescence quantitative kit (Beijing Dining Biotechnology Co., Ltd., Beijing, China); the primers of miR-4796, TLR4, IKKβ, MyD88, NF-κB, U6 (internal reference) and GAPDH (internal reference) (Shenggong Bioengineering Co., Ltd., Shanghai, China); TLR4, IKKβ, MyD88, NF-κB and β-actin primary antibody and goat anti-mouse IgG (CST Inc. Shanghai, China); this was diluted at 1:1000 when used. 

### 4.2. Methods

#### 4.2.1. The Preparation of Ophiopogon Polysaccharide Liposomes

Ophiopogon polysaccharide liposomes (OPL) were prepared by using the reverse-phase evaporation method in the laboratory according to the reference [19]. The weight ratio of soybean phosphatide to Ophiopogon polysaccharide (OP) was 9.5:1, the weight ratio of soybean phospholipid to cholesterol was 8:1, and the volume ratio of chloroform to PBS was 3:1. Firstly, cholesterol and soybean phospholipid were dissolved with chloroform, then PBS contained OP was injected into chloroform. The mixture was homogenized to form stable water in an oil type emulsion. Secondly, the emulsion was evaporated to form a colloid, and then PBS was added to hydrate for 15 min. At this point, the chloroform was evaporated. Thirdly, the mixture was homogenized for 20 min to form a well-proportioned liposome. Finally, the liposome was filtered by using 0.45 and 0.22 μm millipore membrane successively. OPL was obtained. The average particle size, zeta potential, and PDI of OPL were 245.3 nm, −4.56 mV, and 0.326, respectively. The average entrapment rate of OPL was 64.95%. Before use, OPL was diluted to 125, 62.5 and 31.25 μg/mL in complete medium and stored at 4 °C.

#### 4.2.2. Effects of miR-4796 on the Biological Function of KCs Regulated by OPL

##### The Expression of miR-4796 after Transfected with miR-4796 Mimic or Inhibitor by Real-Time PCR Method 

KCs were spread in a six-well cell culture plate at a density of 1 × 10^6^ cells /mL. When the cells grew to 30–40%, miR-4796 mimic or miR-4796 inhibitor with corresponding Negative Control (NC) were separately transfected at the concentration of 50 nM, based on the steps in the instruction of Lipofectamine 2000 transfection reagent. After continuous culture for 36 h, the cells in each well were washed twice with PBS and added 1 mL TRIpure Reagent. Then, total RNA was extracted according to the procedure described in the TRIpure Reagent manual. The extracted total RNA was used to synthesize cDNA by adding reverse transcription primers of miR-4796 or U6 (internal reference) based on the instructions of the 5 × Integrated RT Master Mix reverse transcription kit. The reverse transcription primer sequence of miR-4796 was GTCGTATCCAGTGCAGGGTCCGAGGTATTCGCACTGGATACG ACGTCTAT, and the primer sequence of U6 was GTCGTATCCA GTGCAGGGTCCGAGGTATTCGCACTGGATACGACTTGGTG. Then, as the template cDNA was amplified according to the 2 × Fast qPCR Master Mixture (Green) fluorescence quantitative kit. The primer sequence was as follows: miR-4796, Forward (F): 5′-GCGCGAAGTTGCAGGGT-3′; Reverse (R): 5′-AGTGCAGGGTCCGAGGTATT-3′. U6, F: 5′-CCCTTGAGCAAGGATGGCAT-3′, R: 5′-AGTGCAGGGTCCGAGGTATT-3′. The reaction system was: cDNA for 2 μL, 2 × Master Mix for 10 μL, 0.4 μL for either forward or reverse primers and ddH_2_O for 7.6 μL. The reaction program was 94 °C for 2 min, 94 °C for 15 s, 60 °C for 30 s and 40 cycles. The results of real-time PCR (CFX, Bole Life Medical Products Co., Ltd., Shanghai, China) were analyzed using the 2^−ΔΔCt^ method.

##### miR-4796 Transfection and Treatment of OPL

KCs in a good state were spread in the cell culture plate at a density of 1 × 10^6^/mL. When the cells grew to 30–40%, miR-4796 mimic or miR-4796 inhibitor with corresponding Negative Control (NC) were separately transfected at a concentration of 50 nM, based on the steps in the instruction of Lipofectamine 2000 transfection reagent. After 36 h of transfection and culture, different concentrations of OPL (125–31.25 μg/mL) were added, and the corresponding mimic control (only mimic without OPL) group and inhibitor control (only inhibitor without OPL) group were set. The culturing was continued for 24 h for subsequent experiments.

##### Measurement of NO

After transfection and drug treatment, the supernatant was collected. According to the instruction of nitrite detection kit, the optical absorption density value was detected by a microplate reader (Multiskan FC, Thermo Fisher, Shanghai, China) at a 550 nm wavelength. Then, the concentration of NO was calculated according to the NaNO_2_ standard curve. The calculation formula was as follows: the content of NO (μmoL/mL) = ((determined OD value-blank OD value)/(standard OD value-blank OD value)) × standard concentration (100 μmoL/mL) × sample dilution multiple before testing.

##### Measurement of iNOS

The cells were cultivated for 24 h after transfection with miR-4796 and treated with OPL. The supernatant was collected, the optical absorption density of iNOS was measured at the wavelength of 530 nm, and the content of iNOS was calculated according to the formula in the instructions. The calculation formula was as follows: the content of = ((measured OD value-blank OD value)/38.3 × 10^−6^) × (total volume of reaction solution/sample volume) × (1/(colorimetric path × reaction time))/1000.

##### Cell Scratch Test

The cells were cultivated for 24 h after transfection with miR-4796 and treatment with OPL, and the transfected cells were washed twice with PBS. Then, the cells were lined with 1 mL pipette tips, followed by the removal of the exfoliated cell residue with PBS. The cells continued to be cultured for 24 h under conventional conditions before taking images by microscope (CKX31SF, Olympus Co., Ltd., Tokyo, Japan). The Image J (National Institutes of Health, Bethesda, MD, USA) software was used to analyze the distance between 0 h and 24 h cell scratch boundary. The cell spacing of 24 h was subtracted from the cell spacing of 0 h to obtain the migration distance of the cells cultured for 24 h.

##### Measurement of Phagocytosis of KCs on FITC-Dextran by Fluorescence Staining and Flow Cytometry

The cells were cultivated for 24 h after transfection with miR-4796 and treatment of OPL. After washing twice with PBS, FITC-dextran (1 μg/mL) was added. The cells were incubated at 37 °C for 1.5 h and then washed 3 times with PBS. Finally, one part of the cells was observed and photographed under the fluorescence microscope (ICX41, Ningbo Shunyu Instrument Co., Ltd., Ningbo, China), the other part of cells was resuspended with PBS and filtered with 300 mesh filters, then the cells were measured by flow cytometry (FACSAria III, BD, Franklin Lakes, NJ, USA).

##### Measurement of Phagocytosis of KCs on FITC-OVA by Fluorescence Staining 

The cells were cultivated for 24 h after transfection with miR-4796 and treatment with OPL. FITC-OVA was mixed with DMEM medium and different concentrations of drug groups (125–31.25 μg/mL) and incubated overnight at −20 °C. The medium containing FITC-OVA was added to the transfected cells and the cells were cultured for 24 h. Then, the medium was removed and the cells were washed twice with PBS. The tissue fixative solution was added to each well and fixed at 4 °C for 10 min in darkness. Then, the cells were washed with PBS twice, 3 min each time. DAPI (5 μg/mL) was added to each well and the samples incubated at 37 °C for 15 min. The cells were then washed with PBS twice, 3 min each time. The phagocytosis of OVA-FITC by KCs was observed under the fluorescence microscope.

##### Measurement of Apoptosis by Hoechst 33,258 Staining 

The cells were cultivated for 24 h after transfection with miR-4796 and treatment of OPL. The transfected and treated cells were washed twice with PBS, then 0.1 mL of fixative solution was added and fixed at 4 °C for 1 h. The fixing solution was removed and washed with PBS 3 times. After removing the liquid, 0.1 mL of Hoechst 33,258 staining solution was added and stained for 7 min. After removing the staining solution, cells were washed twice with PBS. Finally, the anti-fluorescence quenched sealing solution was dropped into each well and the cells were placed under an inverted fluorescence microscope for observation and photography.

##### Measuring the Expression Levels of CD14 and MHC II by Flow Cytometry 

The cells were cultivated for 24 h after transfected with miR-4796 and treatment of OPL, the cells were washed twice with PBS. Then the cells were resuspended with PBS, and fluorescently labeled antibodies (PE-CD14, FITC-MHC II) were added at the same time. The cell suspension was left at 4 °C for 30 min in the dark, then filtered with 300 mesh screen, and the expression levels of cell surface molecules CD14 and MHC II were measured by flow cytometry.

##### Measurement of ROS by Fluorescence Staining 

The cells were cultivated for 24 h after transfected with miR-4796 and treatment of OPL, and the cells were washed twice with PBS. After that, DCFH-DA loaded probes were diluted to 1:1000 in serum-free medium, and it was added to the culture plate. The cells were incubated in the cell incubator at 37 °C for 20 min. The cells were washed three times with serum-free cell culture medium to adequately remove DCFH-DA that did not enter the cells. Then the samples were washed with PBS 3 times and photographed under a fluorescence microscope.

#### 4.2.3. The Function of miR-4796 in TLR4-NF-κB Signaling Pathway Regulated by OPL

##### Measuring the mRNA Expression of TLR4, IKKβ, MyD88 and NF-κB by Real-Time PCR

According to the methods of 2.2.1.1, cDNA was obtained and used as the template to amplify by 2 × Fast qPCR Master Mixture (Green) fluorescence quantitative kit. The quantitative primer sequences of TLR4, IKKβ, MyD88, NF-κB and GAPDH (internal reference) were as follows: TLR4, Forward (F): 5′-GCCATCATTATGAGTGCCAATT-3′, Reverse (R): 5′-AGGGATAAGAACGCT GAGAATT-3′; IKKβ, F: 5′-GGCAGAAGAGCGAAGTGGACATC-3′, R: 5′-CCAGCCGTTCAGCCAAGACAC-3′; MyD88, F: 5′-CGGAACTTTTCGATGC CTTTAT-3′, R: 5′-CACACACAACTTAAGCCGATAG-3′; NF-κB, F: 5′-CAAAGACAAAGAGGAAGTGCAA-3′, R: 5′-GATGGAATGTAATCCCACCGT A-3′; GAPDH, F: 5′-GGCAAATTCAACGGCACAGTCAAG-3′, R: 5′-TCGCTCC TGGAAGATGGTGATGG-3′. The reaction program was 94 °C for 2 min, 94 °C for 15 s, 52.5 °C for 30 s, 72 °C for 30 s and 40 cycles. Real-time PCR results were analyzed using the 2^−ΔΔCt^ method.

##### Measuring the Protein Expression of TLR4, IKKβ, MyD88 and NF-κB by Western Blot

KCs were spread in a 12-well cell culture plate at a density of 1 × 10^6^/mL. When the cells grew to 30–40%, miR-4796 mimic or miR-4796 inhibitor with corresponding Negative Control (NC) were separately transfected at a concentration of 50 nM, based on the steps in the instruction of Lipofectamine 2000 transfection reagent. After continuous culture for 36 h, the cells in each well were washed twice with PBS, resuspended with PBS, centrifuged at 3000 r/min for 5 min at 4 °C. Then, 100 μL lysate was added after removing the supernatant. After lysis for 1 h on ice, the samples were centrifuged at 12,000 r/min for 15 min. The protein concentration of the supernatant was determined using the BCA method. Then, the protein concentration was adjusted to the same by the lysate, and 5× protein loading buffer was added to the sample before inactivation at 96 °C for 2 min on the PCR instrument, and the sample was finally stored at −20 °C.

After the preparation of separation glue and concentrated glue, the loading protein was added slowly. The concentrated glue was run at a constant pressure of 80 V for 30 min, and the separated glue was run at a constant pressure of 120 V for 80 min. After electrophoresis, the membrane was transferred at constant pressure of 80 V for 135 min. After the membrane transfer, the PVDF membrane was rinsed in TBST solution and then blocked by shaking for 2.5 h at room temperature or 12 h at 4 °C. The closed membranes were incubated with primary antibody solution overnight at 4 °C by shaking. The PVDF membrane after primary antibody incubation was placed in secondary antibody solution and incubated for 1 h at room temperature or 37 °C by shaking. Finally, the membrane was washed 3 times with TBST, the signal was observed with ECL, and the membrane was exposed on the X-ray film.

#### 4.2.4. Statistical Analysis 

The data were analyzed with statistical software SPSS 21.0 (IBM, Armonk, NY, USA), and the data were expressed as mean ± standard deviation (mean ± SD). Differences in mean values among groups were analyzed using a one-way analysis of variance, and the significance test was performed by LSD. Significant differences were considered at *p* < 0.05, and extremely significant differences were considered at *p* < 0.01(* *p* < 0.05, ** *p* < 0.01).

## 5. Conclusions

After transfected miR-4796, OPL could significantly promote the secretion of NO, iNOS and ROS, cell migration, cell phagocytosis and the expression of CD14 and MHC II. In addition, the mRNA and protein expression levels of TLR4, MyD88 and NF-κB were significantly enhanced. These results indicated that OPL could regulate the immune activity of KCs by regulating miR-4796 and activating the TLR4-NF-κB signaling pathway. However, the target genes of miR-4796 have not been clarified, and the effect of OPL on the target genes of miR-4796 will be further explored in subsequent studies.

## Data Availability

All data are contained within this manuscript.
